# Lateral Window Maxillary Sinus Elevation With Simultaneous Implant Placement: A Step-by-Step Surgical Technique

**DOI:** 10.7759/cureus.107917

**Published:** 2026-04-28

**Authors:** Richard M Cavero, Ines A Camejo, Anyel Torres, Jhonny Gonzalez Ortega, Liliet Dominguez Gonzalez

**Affiliations:** 1 General Practice, Metro Dental Associates, Denville, USA; 2 Dentistry, Smile and Implant Center of Rockland, Nanuet, USA; 3 Dentistry, Advanced Dental Care, Tampa, USA; 4 Dentistry, Boston University School of Medicine, Boston, USA; 5 Dentistry, Agustin Gonzalez DDS, Miami, USA

**Keywords:** bone grafting, dental implant placement, guided bone regeneration, lateral window technique, maxillary sinus augmentation, schneiderian membrane, simultaneous implant placement, sinus floor elevation, sinus lift surgery, subantral augmentation

## Abstract

Maxillary sinus pneumatization following tooth loss can result in reduced vertical bone height in the posterior maxilla, limiting the available bone for dental implant placement. The lateral window sinus elevation technique allows access to the sinus cavity and elevation of the Schneiderian membrane to facilitate bone augmentation.

This technical report presents a step-by-step surgical technique for lateral window maxillary sinus elevation with bone grafting, membrane placement, and simultaneous implant placement. The procedure includes surgical access through a lateral bony window, elevation of the sinus membrane, placement of particulate bone graft material within the subantral space, insertion of a dental implant achieving primary stability, coverage with a resorbable barrier membrane, and tension-free primary closure of the surgical site. This report focuses on intraoperative surgical steps, providing a structured description of the technique for clinicians performing sinus augmentation procedures in the posterior maxilla.

## Introduction

Maxillary sinus pneumatization following tooth loss is a well-recognized physiological process that often results in reduced vertical bone height in the posterior maxilla, limiting the available bone for dental implant placement [1,2]. This anatomical limitation presents a clinical challenge when attempting to achieve adequate primary stability and long-term support for implants.

Maxillary sinus floor elevation using a lateral window approach is a well-established surgical technique to increase subantral bone height and facilitate implant placement. This technique was originally developed by Tatum Jr and later described in the literature by Boyne and James [1,2]. Subsequent consensus reports have contributed to the standardization and clinical application of maxillary sinus augmentation procedures [3]. The lateral window approach allows direct visualization of the Schneiderian membrane, enabling controlled elevation and placement of bone graft material within the sinus cavity [4].

The use of particulate bone graft materials in combination with resorbable membranes has been widely adopted to support guided bone regeneration by stabilizing the graft and preventing soft-tissue infiltration [5,6]. In addition, simultaneous implant placement during sinus augmentation has been reported as a predictable approach when adequate primary stability can be achieved within the residual native bone [7]. This technical report presents a step-by-step surgical approach to lateral window maxillary sinus elevation with bone grafting, membrane placement, and simultaneous implant placement, focusing exclusively on intraoperative clinical procedures.

## Technical report

Maxillary sinus floor elevation was performed using a lateral window approach to increase subantral bone height and facilitate implant placement. This technique follows the principles originally described by Tatum Jr and later reported in the literature by Boyne and James [1,2]. Following administration of local anesthesia, a full-thickness mucoperiosteal flap was elevated in the posterior maxilla to expose the lateral wall of the maxillary sinus. Adequate reflection of the soft-tissues allowed clear visualization of the surgical site. A lateral bony window was created in the lateral wall of the maxillary sinus using rotary instrumentation to allow controlled access to the sinus cavity.

**Figure 1:**
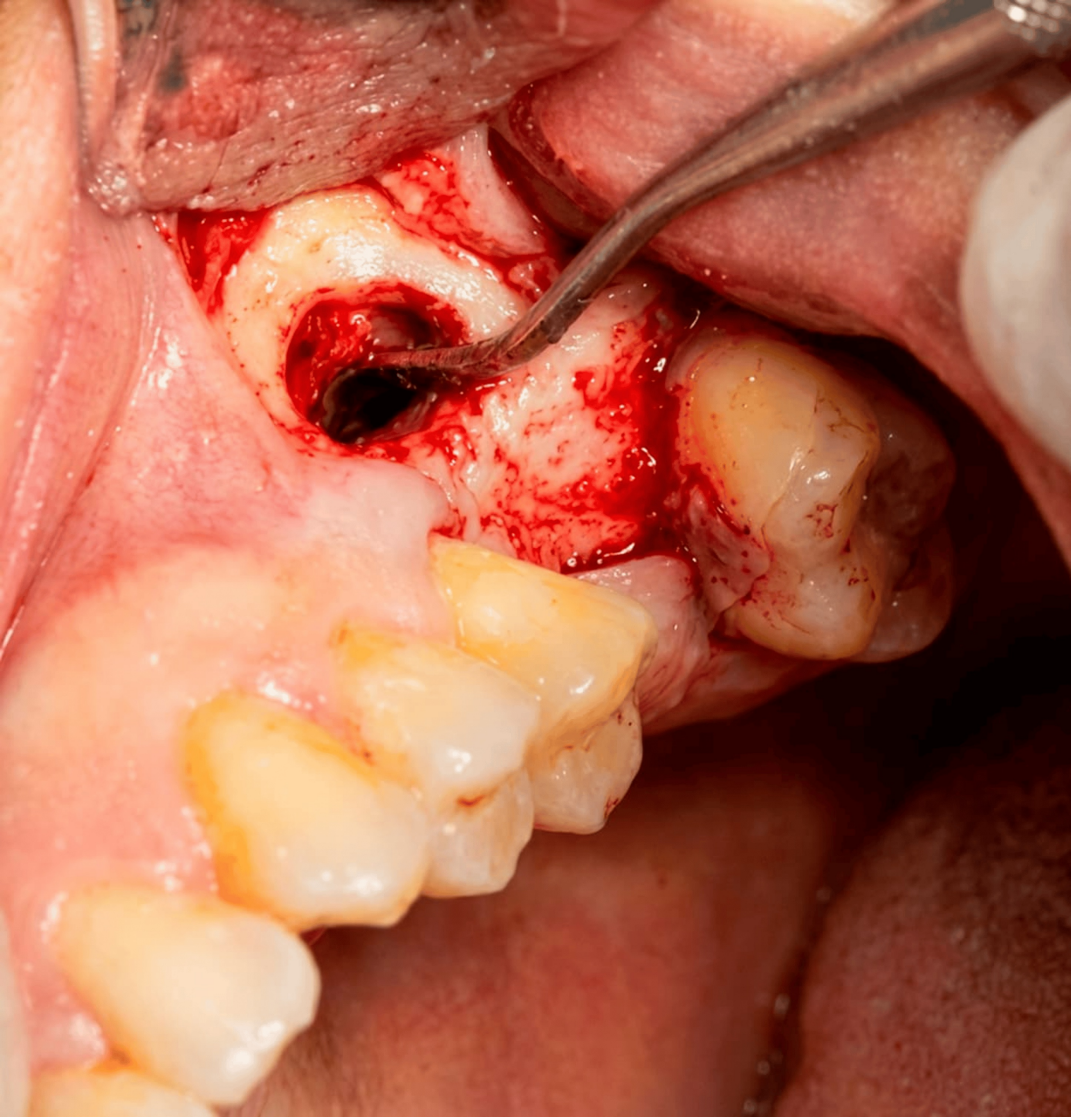
Lateral window osteotomy and initial elevation of the Schneiderian membrane. Intraoperative view of lateral window preparation with initial elevation of the Schneiderian membrane in the posterior maxilla. A surgical instrument is used to carefully detach the sinus membrane from the bony floor while preserving its integrity.

**Figure 2:**
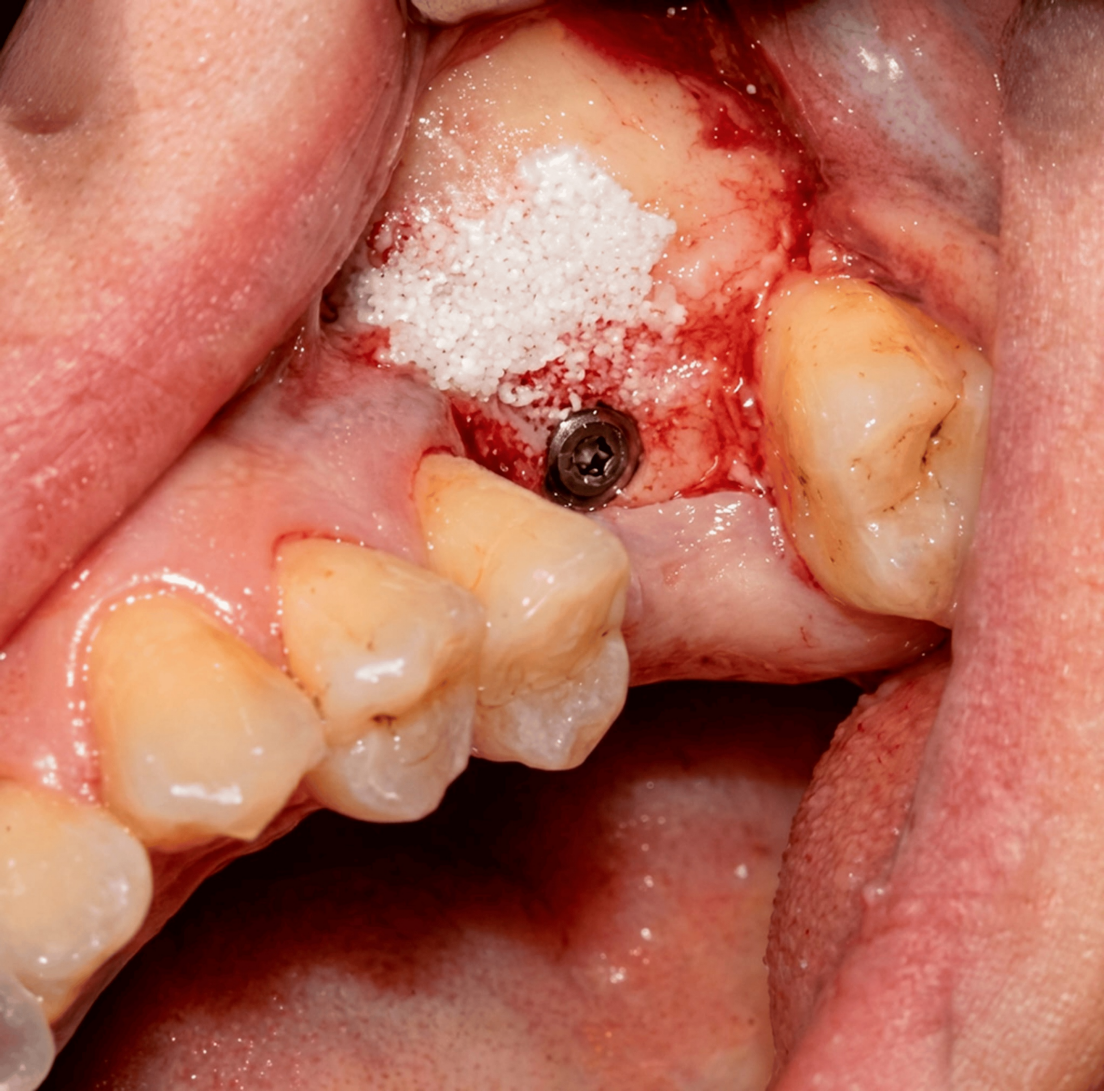
Bone graft placement and simultaneous implant insertion following sinus membrane elevation. Intraoperative view showing placement of particulate bone graft material within the elevated sinus cavity following Schneiderian membrane elevation. A dental implant is simultaneously inserted, achieving primary stability within the residual native bone and surrounded by the grafted material.

**Figure 3:**
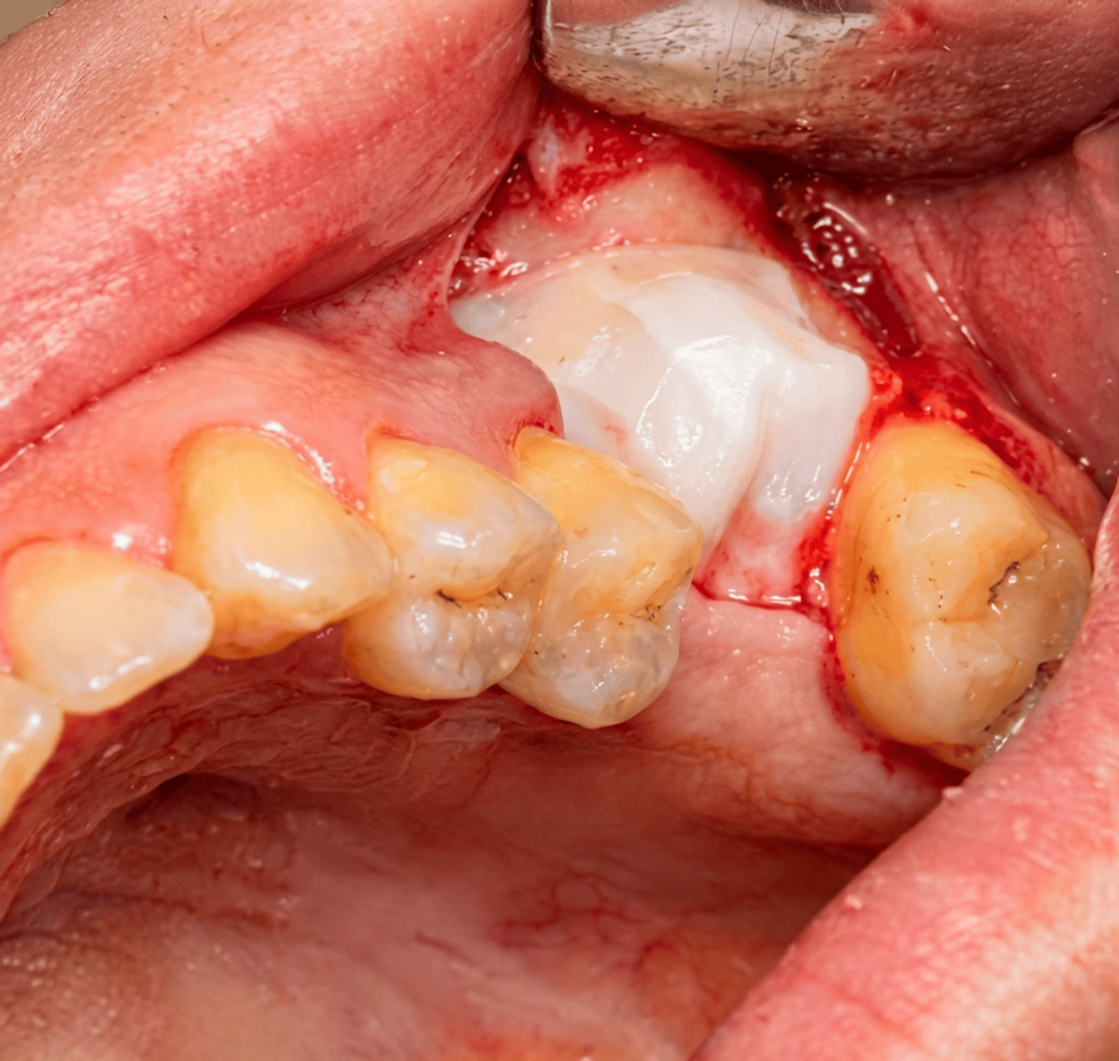
Placement of resorbable membrane over the lateral window following bone grafting and implant insertion. Intraoperative view showing placement of a resorbable collagen membrane over the lateral window to cover the grafted area and implant site. The membrane is positioned to stabilize the particulate bone graft and support guided bone regeneration while preventing soft-tissue infiltration.

**Figure 4:**
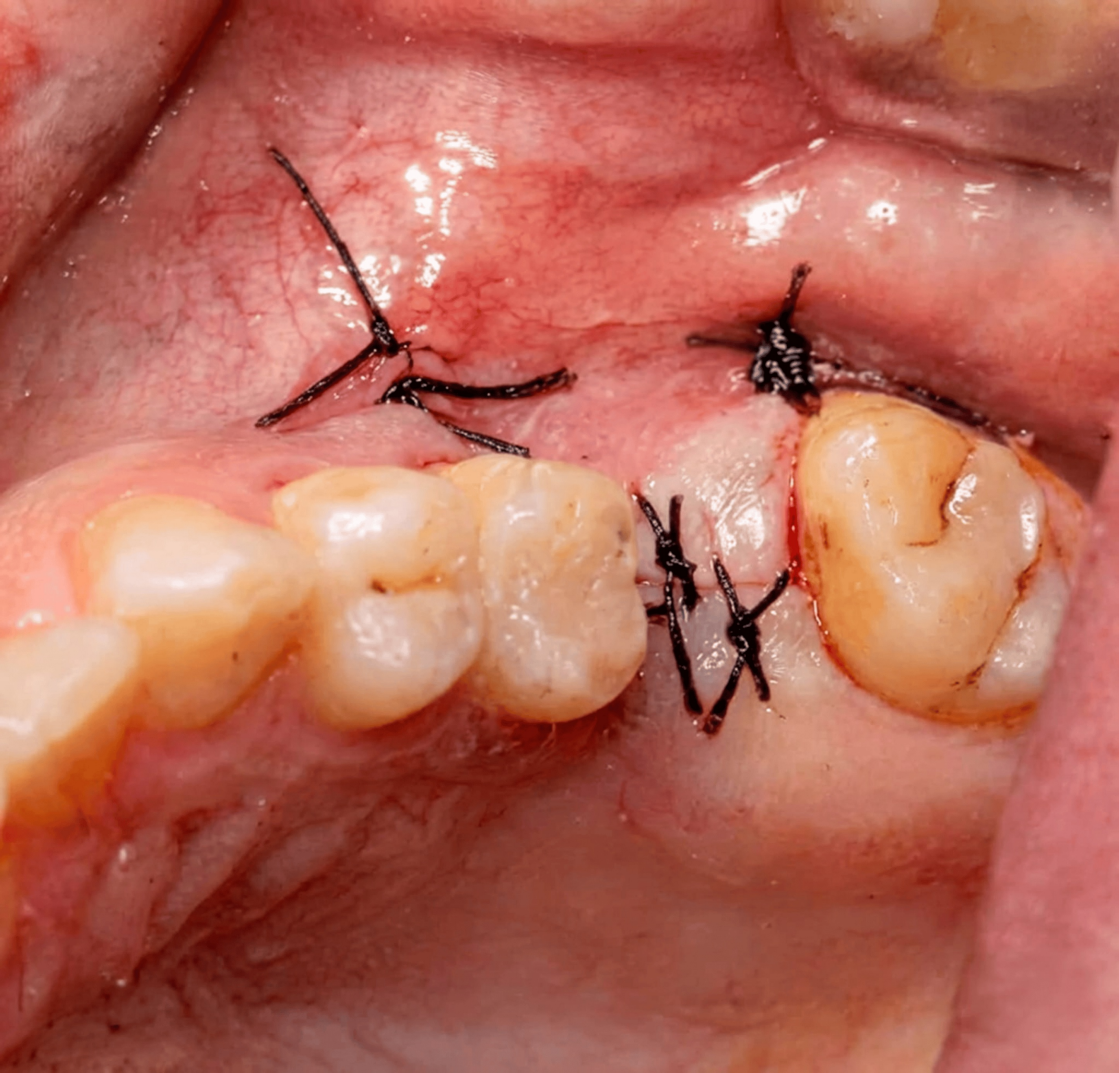
Tension-free primary closure of the surgical site following sinus augmentation and implant placement. Intraoperative view showing final closure of the surgical site with interrupted sutures following lateral window sinus elevation, bone graft placement, membrane coverage, and implant insertion. The flap is repositioned to achieve tension-free primary closure, promoting optimal healing conditions.

Figure 1 demonstrates the intraoperative creation of the lateral window and elevation of the Schneiderian membrane. A surgical instrument was used to carefully detach the membrane from the sinus floor while preserving its integrity. After adequate elevation of the sinus membrane, particulate bone graft material (deproteinized bovine bone xenograft) was placed within the subantral space to augment vertical bone height. Simultaneously, a dental implant was inserted into the prepared osteotomy site, achieving primary stability within the available native bone. Figure 2 illustrates the placement of bone graft material within the sinus cavity in conjunction with implant insertion. Following graft placement and implant insertion, a resorbable collagen membrane was positioned over the lateral window to stabilize the graft material and support guided bone regeneration. Figure 3 demonstrates the membrane positioned over the augmented area. Finally, the mucoperiosteal flap was repositioned and secured using interrupted sutures to achieve tension-free primary closure. Figure 4 shows the final intraoperative view following suturing of the surgical site.

## Discussion

Maxillary sinus pneumatization following tooth loss is a well-documented physiological process that can significantly reduce the available vertical bone height in the posterior maxilla, thereby complicating dental implant placement [1,2]. In such cases, maxillary sinus floor elevation remains a predictable surgical approach for increasing subantral bone volume and facilitating implant therapy.

The lateral window technique, originally developed by Tatum Jr and later described by Boyne and James, is widely regarded as a standard method for sinus augmentation due to the direct visualization it provides of the Schneiderian membrane and the surgical field [1,2]. This direct access allows controlled membrane elevation and precise placement of graft material, reducing the risk of intraoperative complications.

The use of particulate bone graft material within the elevated sinus cavity has been extensively reported to support bone regeneration by providing a scaffold for new bone formation [5]. In addition, the placement of a resorbable membrane over the lateral window is consistent with the principles of guided bone regeneration, helping stabilize the graft material and prevent soft-tissue infiltration [6].

Simultaneous implant placement during sinus augmentation is a widely accepted approach when sufficient primary stability can be achieved within the residual native bone [7]. This approach allows efficient treatment by combining augmentation and implant placement within a single surgical procedure.

This report focuses on the intraoperative surgical protocol, emphasizing a structured, step-by-step approach to lateral window sinus elevation with bone grafting, membrane placement, and implant insertion. Standardization of surgical technique is essential for clinicians aiming to perform predictable sinus augmentation procedures. Several systematic reviews have demonstrated favorable outcomes and predictability of sinus floor elevation procedures with implant placement in the posterior maxilla, and treatment selection in this region has also been guided by proposed clinical decision-making frameworks [4-8].

## Conclusions

The lateral window approach for maxillary sinus floor elevation is a reliable and widely accepted surgical technique for increasing subantral bone height and facilitating implant placement in the posterior maxilla. Careful elevation of the Schneiderian membrane, appropriate placement of bone graft material, use of a resorbable membrane, and tension-free primary closure represent key steps in achieving a structured and reproducible surgical protocol.

This technical report provides a step-by-step intraoperative description of the procedure, offering practical guidance for clinicians performing sinus augmentation. The described approach may contribute to consistent clinical outcomes when applied with appropriate case selection and surgical principles.
